# Association of serum ferritin with metabolic syndrome in eight cities in China

**DOI:** 10.1002/fsn3.1408

**Published:** 2020-02-03

**Authors:** Meichen Wang, Ai Zhao, Ignatius Man‐Yau Szeto, Wei Wu, Zhongxia Ren, Ting Li, Haotian Feng, Peiyu Wang, Yan Wang, Yumei Zhang

**Affiliations:** ^1^ Department of Nutrition and Food Hygiene School of Public Health Peking University Beijing China; ^2^ Department of Social Science and Health Education School of Public Health Peking University Beijing China; ^3^ Inner Mongolia Dairy Technology Research Institute Co. Ltd. Hohhot China; ^4^ Yili Innovation Center Inner Mongolia Yili Industrial Group Co. Ltd. Hohhot China

**Keywords:** insulin resistance, metabolic syndrome, serum ferritin

## Abstract

**Objective:**

This study aims to evaluate the cross‐sectional association of serum ferritin (SF) and the risk of metabolic syndrome (MetS) and its components among adults in eight cities in China.

**Methods:**

Subjects were recruited using a combination of systematic cluster random sampling and purposive sampling in eight cities in China. The sociodemographic characteristics, data of lifestyle factors, self‐reported disease history, and 24‐hr dietary intake were obtained using a validated questionnaire. Anthropometry was performed, and fasting blood was collected to test the SF, fasting blood glucose (FBG), insulin, high‐sensitivity C‐reactive protein (hs‐CRP), triglycerides (TG), and cholesterols. Logistic and linear regression analyses were conducted to investigate the associations, adjusting for age, city level, smoking, drinking, weekly moderate‐to‐vigorous activity, dietary factors, hs‐CRP, and BMI.

**Results:**

Serum ferritin level is positively correlated with total cholesterol, TG, FBG, HOMA‐IR, and hs‐CRP after adjusting for age and BMI. The odds ratio (OR) for MetS in the highest quartile of SF was 2.23 (1.32, 3.77) after adjusting for men, compared with the lowest quartile. An elevated ferritin concentration was significantly related to hypertriglyceridemia (*p* < .001) and elevated glucose (*p* = .013) among men, but not among women. Furthermore, compared with Q1, the OR for insulin resistance in the ferritin Q4 group was 3.08 (1.50, 6.32) among men and 1.96 (1.19, 3.24) among women.

**Conclusion:**

A positive association between elevated SF and MetS and its components including hypertriglyceridemia and elevated glucose was found in multivariate analyses among men, and SF levels are independently associated with IR.

## Graphical abstract

We aim to assess the associations between serum ferritin and components of the metabolic syndrome (and analyses are stratified by gender). The study population was randomly selected based on place of residence, including eight cities in China. In total, 1,659 subjects were included.

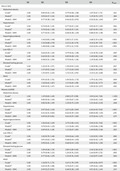

## 1 INTRODUCTION

Iron is required in the human body as a functional component of many proteins which participate in a number of vital biochemical functions, including oxygen transport, energy production, and cytochrome synthesis (Ganz & Nemeth, 2015). Ferritin is known as the form of iron storage, and serum ferritin (SF) levels are widely applied as an indicator of iron status (Ganz & Nemeth, 2015). SF is identified as an important marker of inflammatory disease. Several studies have revealed that elevated SF may be a risk factor for type 2 diabetes mellitus (T2DM; Aregbesola et al., 2018; Chen et al., 2018; Gao et al., 2017), atherosclerosis (Ma et al., 2015; Seo et al., 2015), insulin resistance (IR; Park et al., 2015), and dyslipidemia (Li et al., 2017).

Metabolic syndrome (MetS), a complex pathophysiologic state, is characterized by elevated serum glucose, hypertension, abdominal obesity, and hyperlipidemia (Saklayen, 2018). Several studies have reported that MetS is related to cardiovascular disease (CVD), kidney disease, and T2DM (Beltran‐Sanchez, Harhay, Harhay, & McElligott, 2013). In recent years, many studies have focused on the association between SF and MetS (Chen et al., 2017; Hamalainen, Saltevo, Kautiainen, Mantyselka, & Vanhala, 2012, 2014; Han et al., 2014; Kell & Pretorius, 2014; Ledesma et al., 2015; Shim et al., 2017; Tang et al., 2015), but conflicts still exist among different ethnicities and genders. For instance, a Finland study suggested that higher ferritin levels are associated with hypertriglyceridemia, abdominal obesity, elevated glucose, and lower high‐density cholesterol (HDL‐C), but not with MetS (Hamalainen, Saltevo, Kautiainen, Mantyselka, & Vanhala, 2012). Nevertheless, a significant association was found between SF and MetS in Korean adults (Shim et al., 2017). In China, some studies have concentrated on exploring the association between SF and MetS. The Fangchenggang Area Males Health and Examination Survey (FAMHES) conducted in Guangxi discussed this association in the Chinese male population (Tang et al., 2015). Another study reported that SF levels are independently associated with MetS and IR in the Beijing area (Chen et al., 2017). A study in the Yi ethnic group showed that the risk of MetS was significantly higher in female subjects who had elevated ferritin levels (Wei et al., 2015). One study from the China Health and Nutrition Survey (CHNS) found the association among men after adjustment (Han et al., 2014), while another reported the associations among men and women (Li, Wang, Luo, Li, & Xiao, 2013). Some previous studies were conducted in one area of China, and their results were still inconsistent. Importantly, most of these studies did not consider the potential effects of dietary factors, physical activities, and inflammatory status.

In the present study, we collected clinical parameters and field investigation data from eight cities in China, and the confounding factors were controlled very well. We aim to evaluate the independent association between SF and the risk of MetS and its components among adults in eight cities in China, and to explore whether there are gender differences.

## 2 MATERIALS AND METHODS

### 2.1 Subjects

The data used in the present study were extracted from the Chinese Urban Adults Diet and Health Study, a cross‐sectional survey conducted from March to July 2016. The sampling methods were described elsewhere (Zhao et al., 2017). Briefly, subjects were recruited using a combination of systematic cluster random sampling and a purposive sampling method. Firstly, we selected two communities in each first‐tier city (Beijing and Guangzhou) and one community in each non‐first‐tier city (Chengdu, Chenzhou, Jilin, Lanzhou, Wuhu and Xuchang). Secondly, a random sampling method was used to recruit participants aged 18–75 years based on resident registration. Lastly, at least 60, 60 and 50 residents were included for three age groups (18–44, 45–64 and >65 years), respectively. Participants were excluded if they had a known mental disease, infectious disease, memory problem, or if women were pregnant or lactating. A total of 1,739 individuals aged 18 years or older were included. In the present analysis, participants were excluded if they had missing data on SF, fasting blood glucose (FBG), blood pressure (BP), or body mass index (BMI). The final analysis consisted of 1,659 individuals. All participants provided written informed consent, and the study was approved by the Medical Ethics Research Board of Peking University (No. IRB00001052‐15059).

### 2.2 Data collection and variable definitions

The sociodemographic characteristics (e.g., age and gender), data of lifestyle factors (smoking status, alcohol intake, and physical activity), self‐reported disease history (hypertension, diabetes mellitus, and hyperlipidemia), and 24‐hr dietary intake before the investigation day were obtained by trained interviewers through a validated questionnaire. In the analysis, cigarette smoking status was grouped as never or current/former smoker, and if participants self‐reported alcohol intake in the past month, they were defined as an alcohol consumer. Weekly moderate‐to‐vigorous physical activity levels were grouped as <0.5, 0.5–3.9 or >4.0 hr/week, and cities were grouped as first‐tier or non‐first‐tier cities according to their economic status. The 24‐hr dietary intake data were used to calculate energy and macronutrient intake according to the *Chinese Food Composition Table* (Yang, 2004, 2009). BP, height, weight, and waist circumference (WC) were collected by trained interviewers or professional nurses in the field. BP was measured twice using an electronic BP monitor (Omron HEM‐7124). BMI was calculated as weight (kg) divided by height squared (m^2^).

### 2.3 Laboratory measurements

A total of 10 ml of fasting venous blood was drawn from participants and centrifuged and separated into plasma, serum, and red blood cells, then transported to the local hospital and stored in a freezer. A total of 1,696 blood samples were collected. Finally, samples were transported to Beijing and stored at −80°C in freezers. All samples were analyzed by the Lawke Health Laboratory with strict quality control. SF was measured by the chemiluminescence method, and FBG was determined by the glucose oxidase method. Insulin level was analyzed by enzyme‐linked immunosorbent assay (ELISA), and serum triglycerides, cholesterol, and lipoprotein cholesterol were measured by using the glycerol oxidase 4‐chloro acid method, cholesterol oxidase aminoantipyrine phenol method, and direct method, respectively. In addition, we also used a turbidimetric immunoassay to detect serum hypersensitive C‐reactive protein (hs‐CRP).

### 2.4 Diagnostic criteria of disease

The diagnostic criteria for MetS used were according to the 2017 version of the *Guidelines for the Prevention and Treatment of Type 2 Diabetes in China (The 2017 version)* and a joint multinational interim statement (Alberti et al., 2009) as the presence of three or more of the following risk factors: WC >90 cm in men or >85 cm in women; serum triglyceride concentration >1.7 mmol/L or specific treatment for this lipid abnormality; HDL‐cholesterol concentration <1.0 mmol/L in men or <1.3 mmol/L in women or specific treatment; BP 130/85 mmHg or greater or treatment of previously diagnosed hypertension; or serum glucose concentration of ≥5.6 mmol/L or previously diagnosed T2DM. The Homeostasis Model Assessment (HOMA) index was calculated using the equation: HOMA‐index ＝ [Insulin (mIU/L) × FBG (mmol/L)]/22.5, and the highest quartile of HOMA was defined as IR.

### 2.5 Statistical analysis

The analyses were stratified by sex. Categorical variables were described by percentages and proportion and compared using the chi‐squared test. Normally and non‐normally distributed continuous variables were reported by geometric means and standard deviations, and medians and interquartile ranges (25th and 75th percentages), respectively. Student's *t* test or the Mann–Whitney test were used when continuous variables were compared. Partial correlation coefficients (adjusted for age and BMI) between SF and risk factors of MetS components, fasting insulin, HOMA‐IR, and hs‐CRP were calculated in the overall studied population. Skewed variables (SF, FBG, fasting insulin, HOMA‐IR, and hs‐CRP) were all normalized by logarithmic transformation (ln) and were treated as continuous variables in partial correlation.

Logistic regression models were applied to analyze the relationship between SF and MetS as well as its components. Participants were categorized into quartiles based on the distribution of SF and we took the lowest quartile as the reference in both genders. The crude model was the unadjusted model. Model 1 was adjusted for age, city level, smoking, drinking, activity, dietary factors (total energy intake, fat intake, and protein intake), and hs‐CRP. All data management and statistical analyses were performed with SPSS version 20.0 (International Business Machines Corporation). Statistical tests were performed two‐tailed, and a *p*‐value <.05 was considered statistically significant.

## 3 RESULTS

### 3.1 Characteristics of studied population

A total of 1,659 participants (561 men and 1,098 women) were included, and their demographic characteristics and lifestyle factors were stratified by gender as shown in Table 1. The rates of cigarette smoking and alcohol consumption were significantly higher in men. BMI, WC, BP, dietary energy, macronutrient intake, FBG, HbA1c, SF, total cholesterol, TG concentration, and HOMA‐IR were all higher in men, whereas men had lower HDL‐C, fasting insulin, and hs‐CRP concentrations than women. There was no significant difference in the duration of weekly moderate‐to‐vigorous activity and LDL‐C concentration.

**Table 1:** Characteristics of studied population, segregated by gender (mean values and standard deviations; medians and interquartile ranges [IQR])

	All (*n*: 1659)		Men (*n*: 561)		Women (*n*: 1,098)		*p* Value
	Mean	*SD*	Mean	*SD*	Mean	*SD*	
Sociodemographic characteristics							
Age (years)	50.16	17.42	54.39	16.51	48.00	17.48	<.001
City level (first‐tier cities, %)	37.67		32.62		40.26		.002
Lifestyle							
Cigarette smoker (%)	33.39		62.39		3.46		<.001
Alcohol consumer (%)	27.55		49.55		16.3		<.001
Weekly moderate‐to‐vigorous activity (hr)							.208
Median	1.00		1.17		1.00		
IQR	0–4.50		0–5.17		0–4.00		
Anthropometry							
BMI (kg/m^2^)	23.84	3.55	24.58	3.75	23.46	3.38	<.001
WC (cm)	82.23	10.7	86.6	10.44	79.99	10.13	<.001
SBP (mmHg)	124.13	19.05	129.6	17.49	121.34	19.21	<.001
DBP (mmHg)	79.11	22.47	83.24	26.47	77.01	19.8	<.001
Diet							
Energy (KJ/day)	7,030.29	3,231.93	8,041.56	3,437.53	6,513.61	2,994.11	<.001
Dietary protein (g/day)	56.87	35.39	66.11	37.29	52.15	33.42	<.001
Dietary fat (g/day)	60.84	35.22	66.84	37.18	57.78	33.79	<.001
Dietary carbohydrate (g/day)	234.22	130.11	267.61	137.08	217.15	123.01	<.001
Biochemistry							
FBG (mmol/L)	5.55	1.53	5.79	1.75	5.42	1.38	<.001
Fasting insulin (pmol/L)							
Median	55.09		47.43		58.54		<.001
IQR	35.87–77.73		30.37–71.67		39.42–81.42		
Serum ferritin (pmol/L)	4.71	0.96	4.61	0.95	4.75	0.97	.004
Total cholesterol (mmol/L)							
Median	1.24		1.36		1.19		<.001
IQR	0.88–1.84		0.97–2.04		0.85–1.75		
HDL‐cholesterol (mmol/L)	1.4	0.35	1.26	0.33	1.47	0.34	<.001
LDL‐cholesterol (mmol/L)	2.85	0.83	2.81	0.81	2.87	0.84	.174
TG (mmol/L)							
Median	0.67		0.75		0.65		<.001
IQR	0.31–1.41		0.43–1.45		0.27–1.36		
hs‐CRP (mg/L)							
Median	2.05		1.82		2.17		<.001
IQR	1.32–2.98		1.14–2.77		1.45–3.08		
HOMA‐IR							
Median	2.15		4.00		1.33		<.001
IQR	0.92–4.12		2.48–6.01		0.65–2.81		

Abbreviations: BMI, body mass index; DBP, diastolic blood pressure; FBG, fasting blood glucose; HOMA‐IR, Homeostasis Model Assessment for Insulin Resistance; hs‐CRP, high‐sensitivity C‐reactive protein; SBP, systolic blood pressure; WC, waist circumference.

### 3.2 Partial correlation coefficients between SF and risk factors of MetS

The partial correlation coefficients (adjusted for age) between SF and BMI were 0.141 (*p* = .001) in men and 0.027 (*p* = .363) in women. The partial correlation coefficients adjusted for age and BMI between SF and the indicators of MetS are shown in Table 2. Overall, SF was significantly positively associated with total cholesterol, TG, FBG, HOMA‐IR, and hs‐CRP; these associations could also be observed for different genders. Furthermore, SF was significantly correlated with WC, systolic BP, and HDL‐C in the overall study sample but not specified for different genders. The significant correlation between SF and fasting insulin was only found in women. There was no statistically significant correlation between SF and other indicators, including diastolic BP and LDL‐C.

**Table 2:** Partial correlation coefficients (adjusted for age and BMI) between SF and risk factors of MetS in the studied population, segregated by gender

	All (*n*: 1,659)		Men (*n*: 561)		Women (*n*: 1,098)	
	*r*	*p* Value	*r*	*p* Value	*r*	*p* Value
WC	.101	<.001	−.056	.189	−.041	.18
SBP	.083	.001	−.016	.709	.015	.624
DBP	.018	.474	.006	.898	−.044	.153
Total cholesterol	.093	<.001	.176	<.001	.12	<.001
TG	.166	<.001	.167	<.001	.114	<.001
HDL‐C	−.141	<.001	−.008	.845	−.015	.627
LDL‐C	.044	.075	.063	.14	.058	.057
lnFBG	.123	<.001	.098	.022	.12	<.001
lnFasting insulin	.042	.094	.244	<.001	.108	<.001
lnHOMA‐IR	.049	.046	.242	<.001	.115	<.001
lnhs‐CRP	.162	<.001	.119	.005	.179	<.001

Abbreviations: DBP, diastolic blood pressure; FBG, fasting blood glucose; HOMA‐IR, Homeostasis Model Assessment for Insulin Resistance; hs‐CRP, high‐sensitivity C‐reactive protein; SBP, systolic blood pressure; WC, waist circumference.

### 3.3 Multivariate analysis for MetS and its components

Logistic regression models were applied to evaluate the association between SF and MetS and its components according to sex‐specific quartiles of SF in Table 3. The crude model was the unadjusted model. Model 1 was adjusted for age, city level, smoking, drinking, activity, dietary factors (total energy intake, fat intake, and protein intake), and hs‐CRP.

**Table 3:** Adjusted odds ratios (95% CIs) for MetS and its components according to SF quartile by gender (odds ratios [95% confidence intervals])

	Q1	Q2	Q3	Q4	*p*_trend_
Men (*n* 561)					
Abdominal obesity					
Crude^a^	1.00	0.84 (0.52, 1.35)	0.79 (0.48, 1.28)	1.07 (0.67, 1.71)	.561
Model1^b^	1.00	0.93 (0.57, 1.54)	0.81 (0.48, 1.35)	1.19 (0.72, 1.97)	.384
Model1 + BMI	1.00	0.77 (0.38, 1.52)	0.46 (0.23, 0.92)	0.52 (0.26, 1.04)	.079
Hypertension					
Crude^a^	1.00	0.73 (0.45, 1.19)	0.77 (0.47, 1.25)	0.92 (0.57, 1.50)	.906
Model1^b^	1.00	0.74 (0.42, 1.30)	0.65 (0.37, 1.14)	0.87 (0.49, 1.55)	.423
Model1 + BMI	1.00	0.77 (0.44, 1.35)	0.68 (0.38, 1.20)	0.88 (0.50, 1.58)	.943
Hypertriglyceridemia					
Crude^a^	1.00	1.43 (0.83, 2.48)	2.00 (1.17, 3.41)	3.68 (2.19, 6.20)	<.001
Model1^b^	1.00	1.54 (0.88, 2.70)	2.15 (1.24, 3.74)	4.14 (2.39, 7.19)	<.001
Model1 + BMI	1.00	1.48 (0.82, 2.68)	1.99 (1.12, 3.55)	3.63 (2.04, 6.45)	<.001
Low HDL‐C					
Crude^a^	1.00	0.62 (0.35, 1.10)	0.79 (0.46, 1.38)	1.31 (0.78, 2.18)	.069
Model1^b^	1.00	0.63 (0.35, 1.14)	0.85 (0.48, 1.51)	1.42 (0.82, 2.46)	.040
Model1 + BMI	1.00	0.58 (0.31, 1.05)	0.75 (0.42, 1.36)	1.19 (0.68, 2.09)	.155
Elevated fasting glucose					
Crude^a^	1.00	1.22 (0.74, 1.97)	1.50 (0.92, 2.44)	1.58 (0.98, 2.55)	.067
Model1^b^	1.00	1.57 (0.92, 2.66)	1.77 (1.04, 3.03)	2.30 (1.34, 3.95)	.005
Model1 + BMI	1.00	1.55 (0.91, 2.64)	1.72 (1.01, 2.95)	2.13 (1.23, 3.68)	.013
MetS					
Crude^a^	1.00	0.91 (0.55, 1.51)	1.04 (0.63, 1.72)	1.79 (1.10, 2.91)	.004
Model1^b^	1.00	1.10 (0.65, 1.87)	1.17 (0.69, 1.98)	2.23 (1.32, 3.77)	.001
Model1 + BMI	1.00	0.84 (0.42, 1.67)	0.66 (0.33, 1.34)	1.05 (0.53, 2.07)	.652
Women (n1098)					
Abdominal obesity					
Crude^a^	1.00	1.19 (0.83, 1.69)	2.80 (1.97, 3.98)	4.94 (3.44, 7.10)	＜.001
Model1^b^	1.00	0.83 (0.56, 1.24)	1.01 (0.67, 1.54)	1.02 (0.65, 1.62)	.669
Model1 + BMI	1.00	0.85 (0.48, 1.49)	1.33 (0.75, 2.35)	1.21 (0.65, 2.25)	.468
Hypertension					
Crude^a^	1.00	0.97 (0.65, 1.43)	2.67 (1.85, 3.84)	6.43 (4.43, 9.35)	<.001
Model1^b^	1.00	0.51 (0.31, 0.83)	0.63 (0.39, 1.03)	0.80 (0.48, 1.34)	.676
Model1 + BMI	1.00	0.49 (0.29, 0.81)	0.61 (0.37, 1.00)	0.75 (0.44, 1.27)	.679
Hypertriglyceridemia					
Crude^a^	1.00	1.06 (0.66, 1.69)	2.75 (1.80, 4.21)	5.04 (3.33, 7.62)	<.001
Model1^b^	1.00	0.74 (0.44, 1.23)	1.11 (0.68, 1.81)	1.43 (0.86, 2.38)	.019
Model1 + BMI	1.00	0.69 (0.41, 1.18)	1.12 (0.67, 1.85)	1.40 (0.83, 2.36)	.023
Low HDL‐C					
Crude^a^	1.00	1.02 (0.70, 1.48)	0.96 (0.66, 1.40)	1.32 (0.92, 1.90)	.085
Model1^b^	1.00	0.91 (0.62, 1.34)	0.68 (0.44, 1.03)	0.82 (0.52, 1.29)	.639
Model1 + BMI	1.00	0.90 (0.61, 1.34)	0.68 (0.44, 1.05)	0.82 (0.52, 1.30)	.642
Elevated fasting glucose					
Crude^a^	1.00	1.09 (0.68, 1.74)	2.89 (1.89, 4.42)	5.42 (3.58, 8.19)	<.001
Model1^b^	1.00	0.79 (0.48, 1.31)	1.24 (0.76, 2.01)	1.64 (0.99, 2.72)	.005
Model1 + BMI	1.00	0.77 (0.46, 1.29)	1.25 (0.76, 2.04)	1.62 (0.97, 2.69)	.006
MetS					
Crude^a^	1.00	1.10 (0.71, 1.71)	2.67 (1.78, 3.99)	6.04 (4.06, 8.97)	<.001
Model1^b^	1.00	0.65 (0.39, 1.08)	0.76 (0.47, 1.25)	1.09 (0.65, 1.80)	.088
Model1 + BMI	1.00	0.49 (0.27, 0.91)	0.68 (0.38, 1.20)	0.91 (0.51, 1.64)	.214

Abbreviations: BMI, body mass index; MetS, metabolic syndrome.

a Crude: unadjusted.

b Model 1: adjusted for age, city level, smoking, drinking, weekly moderate‐to‐vigorous activity, dietary factors (total energy intake, fat intake, and protein intake), and hs‐CRP.

Odds ratios (ORs) for abdominal obesity, hypertension, hypertriglyceridemia, elevated fasting glucose, and MetS in the highest quartile group (Q4) of SF were statistically significantly increased in the crude model among women, but the association was weakened or not significant after adjustment in model 1. ORs for MetS and its components in Q4 of SF were not statistically significant after further adjustment for parameters of BMI among women.

In men, no significant association between SF and abdominal obesity, hypertension and low HDL‐C was observed. After adjusting for age, city level, smoking, alcohol consumption, activity, dietary factors, and hs‐CRP, the association between SF and MetS was statistically significant. With an increase in SF quartile group, the risk of hypertriglyceridemia showed an improvement, and this trend still existed in model 1 (OR 4.14, 95% CI: 2.39, 7.1; *p*_trend_ < .001) and the further adjusted model (OR 3.63, 95% CI: 2.04, 6.45; *p*_trend_ < .001), while the ORs for elevated glucose were 2.30 (95% CI: 1.34, 3.95; *p*_trend_ = .005) and 1.79 (95% CI: 1.10, 2.91; *p*_trend_ = .013) in model 1 and the further adjusted model, respectively.

### 3.4 Multivariate analysis for IR

The OR for the risk of IR in the highest quartile group of SF levels was significantly increased in the unadjusted model (OR 3.45, 95% CI: 1.83, 6.48; *p*_trend_ < .001); however, after adjustment, the OR for the risk of IR decreased to 3.08 (95% CI: 1.50, 6.32; *p*_trend_ = .002) for further adjustment among men, while the OR for IR was decreased from 2.78 (95% CI: 1.89, 4.09, *p*_trend_ < .001) to 1.96 (95% CI: 1.19, 3.24, *p*_trend_ < .001) among women.

## 4 DISCUSSION

The results of the present cross‐sectional study suggest that SF level is independently associated with IR in men as well as in women. Furthermore, we found a positive association between elevated SF and MetS and its components, including elevated glucose and hypertriglyceridemia, after adjustment for possible confounders in covariance analyses among men rather than women. However, the association of MetS was not significant after adjustment for BMI.

Several previous studies analyzed the association between SF and metabolic disease stratified by gender. In the Korean National Health and Nutrition Examination Survey (KNHANES; Shim et al., 2017), the highest SF quartile exhibited a 1.62‐fold (95% CI: 1.28–2.12) increased risk of MetS in males and a 1.36‐fold (95% CI: 1.09–1.69) increased risk in females compared with the lowest quartile after adjustment. In CHNS (Han et al., 2014), elevated SF levels were significantly related with a higher risk of MetS (OR 5.46, 95% CI: 3.17, 9.39) among men after adjusting for age, region, smoking, drinking, and dietary factors, but not among women. A strong gender difference in association between SF and MetS was also found in the present study. The influence of sex may be related to the characteristics of the population sampled and differences in iron depletion (Han et al., 2014).

With respect to the association between SF and the components of MetS, the findings in different studies were inconsistent and inconclusive. The Aragon Workers’ Health Study (AWHS; Ledesma et al., 2015) suggested that SF is significantly associated with MetS defining criteria (central obesity, hypertriglyceridemia, hypertension, and hyperglycemia), especially central obesity and hypertriglyceridemia among Spanish male adults. A follow‐up study conducted in Finland (Hamalainen, Saltevo, Kautiainen, Mantyselka, & Vanhala, 2014) reported that decreasing levels of SF indicate resolving hypertriglyceridemia and hyperglycemia. The 2007–2008 KNHANES (Kang, Linton, & Shim, 2012) also found an association of SF with high TG and glucose concentrations in men and women, respectively. CHNS (Han et al., 2014) found that an elevated concentration of ferritins was significantly related to a higher risk of the five components of MetS among men, but not among women. A recent meta‐analysis (Suarez‐Ortegon et al., 2018) indicated that high TG and glucose are the components more strongly associated with SF. In our study, hypertriglyceridemia and elevated glucose were associated with SF among men. ORs for hypertriglyceridemia and elevated glucose in Q4 of SF were not statistically significantly increased, but there was a linear upward trend (*p* < .05) among women. Enlarging the sample size in future studies may show an association. No association between SF and other components of MetS was observed.

Body mass index is considered to be an anthropometric predictor of cardiovascular disease (Reis et al., 2015), and it was correlated with SF in males. Obesity, identified by BMI, is also related to elevated SF (Han et al., 2014; Park et al., 2014). Thus, adjusting for BMI allows investigation of whether any association exists independently of obesity. In the present study, SF level was positively correlated with total cholesterol, TG, FBG, HOMA‐IR, and hs‐CRP after adjusting for age and BMI, but the association of MetS did not reach the level of significance after adjustment for BMI. A meta‐analysis (Suarez‐Ortegon et al., 2018) also found that the meta‐regression for ferritin–MetS association identified weaker associations when the studies adjusted for BMI, but the role of adjustment for BMI in evaluating confounding factors should be considered. FAMHES (Tang et al., 2015) has not yet found an association between SF and MetS after adjusting for BMI among Chinese males. There may be strong associations between BMI and the components of MetS, such as WC. Therefore, consideration of whether or not to adjust for BMI when evaluating confounding factors and probable underlying mechanisms should be made in future research.

The potential pathophysiology underlying SF levels and MetS is still not clear. Some explanations are related to IR and oxidative stress caused by excess body iron (Fargion et al., 2005). Recently, a new hypothesis, called the Iron Dysregulation and Dormant Microbes (IDDM) hypothesis, has been proposed and considers IDDM to underpin a host of metabolic diseases (Kell & Pretorius, 2018). Iron metabolism in the body, consisting of iron conservation and recycling, is controlled by hepcidin. Meanwhile, hepcidin is homeostatically regulated by iron and erythropoietic activity (Ambachew & Biadgo, 2017). Therefore, iron in a healthy person is in a state of metabolic balance. A multitude of factors that release free iron into the bloodstream leading to raised SF can initiate iron dysregulation, such as nutritional stress (Schaffer, 2016) and oxidative stress (Nanba et al., 2016). In high iron conditions, hepcidin alone is not sufficient to regulate iron homeostasis (Parmar, Davis, Shevchuk, & Mendes, 2017). Iron dysregulation and elevated SF might result in cell death and microbial reactivation, then inflammagens such as lipopolysaccharide (LPS) and lipoteichoic acid (LTA) will be produced. These inflammagens can damage pancreatic β‐cells and cause IR and metabolic disorders (Kell & Pretorius, 2018). Metabolic disorders may also influence iron dysregulation. Consequently, SF levels are associated with metabolic diseases, but it is hard to be certain about the exact sequence of causality. Prospective studies should be conducted to confirm whether elevated SF predicts MetS and its components or is just a biomarker of metabolic disturbance.

The present study was conducted using strict procedures, including the collection of questionnaires, body measurements, and blood samples in eight cities in China. The association between MetS and its components was analyzed after adjusting for possible confounders, including physical activity, dietary factors, inflammation markers, and BMI. Meanwhile, some limitations must be acknowledged. We did not identify a genetic cause or hepcidin levels underlying SF. In addition, as this is a cross‐sectional study, some recall biases might exist and causation cannot be inferred, and some confounding information such as family history of disease was not collected.

## 5 CONCLUSIONS

In conclusion, we found a positive association between elevated SF and MetS and its components including elevated glucose and hypertriglyceridemia after adjustment for possible confounders in multivariate analysis among men rather than women. SF levels were independently associated with IR in men and women, respectively. Further studies should be conducted to investigate the pathophysiologic mechanisms and to confirm whether elevated SF predicts metabolic disease.

## CONFLICT OF INTEREST

All the authors declare no conflict of interest.

## ETHICAL STATEMENT

The study conforms to the Declaration of Helsinki, US and does not embrace any human or animal testing. Written informed consent from every participant had been obtained and documented, and the study's protocols and procedures were ethically reviewed and approved by the Medical Ethics Research Board of Peking University (No. IRB00001052‐15059).
